# On the Nature of Croupous Inflammations, with Special Reference to the Puerperium

**Published:** 1889-07

**Authors:** Charles G. Stockton

**Affiliations:** Professor of the Principles and Practice of Medicine and Clinical Medicine, Medical Department of the University of Buffalo; 278 Franklin Street


### *ON THE NATURE OF CROUPOUS INFLAMMATIONS, WITH SPECIAL REFERENCE TO THE PUERPERIUM.*[1]

By CHARLES G. STOCKTON, M. D.,

Professor of the Principles and Practice of Medicine and Clinical Medicine, Medical Department of the University of Buffalo.

The mucous surfaces of the body, under certain conditions, take on what we know as catarrhal inflammations, which may assume various degrees of intensity.

When these inflamed structures become excessively hyperemic, when there appears a deep, livid color, much swelling, and the free surfaces are covered with a fibrinous exudate, which replaces the epithelium, and either lies upon or involves the submucous tissues—an exudate that may be thin and semi-transparent, or of considerable thickness and toughness, and opaque—a pseudo-membrane arising from the fibrin-forming elements of the part, and the fibrinogen of the blood, acting under the stimulus of a special ferment, we have to deal with what is known as a croupous inflammation.

This form of inflammation may arise from traumatism, from the action of chemicals, from the irritants accompanying certain diseases. Thus, we occasionally observe it after injury to a part from long-continued pressure, after the application of carbolic acid, or under irritation of scarletinal, rubeolar, or pyemic poisons. It occurs in the course of, and makes up a part of, the ordinary pathology of acute pericarditis, pleuritis, and lobar pneumonia. It is seen in that rather rare affection called croupous bronchitis, in which disease certain bronchi may be partly or wholly occluded by fibrinous growth for days together, to be followed

---

1. Read before the Buffalo Obstetrical Society, April 26, 1889.

by a similar development in adjoining bronchi, and so on, the affection assuming almost a chronic character, at length proving fatal, or ending after a slow convalescence, in which there has occurred numerous relapses.

Amongst the special causes of croupous inflammations, one of the most frequent is diphtheria. It was at one time believed that the fibrinous exudation of this disease was a peculiar one; that there were histological differences that made it possible to distinguish the diphtheritic from other croupous pseudo-membranes; but it is now generally admitted that, in the language of Dr. Prudden, this is "a distinction which does not distinguish," that the fibrinous exudate attending other diseases may involve as deep-lying tissues, become as rapidly granular, and in other ways behave as does that of true diphtheria.

To differentiate between the pseudo-membranous growth, then, we must look for further evidence than those afforded by the history of the growth, in certain instances. In other words, a croupous inflammation has certain characteristics common to it, no matter what the exciting cause, but conducting itself in obedience to the intensity of the irritation—sometimes slow in degeneration; sometimes rapidly becoming necrotic; sometimes covering extensive infiltration of subjacent tissues; sometimes only a superficial disturbance; but always a croupous inflammation, whether from chemical, traumatic, or infectious origin.

It may be remarked that these views uphold the theory of the duality of croupous and diphtheritic pseudo-membranes. Such is not the intention of the writer; but it is intended to show the unity of croupous exudates in general, at the same time maintaining that such exudates come from a multiplicity of causes; that the appearance of such growths is a symptom belonging to distinct affections, varying widely in nature; and that the life history of the fibrinous growth varies materially in each of these several distinct affections, depending always upon the intensity and the nature of the irritation in the given case, and the natural vital resistance in the given subject.

It is held that the mere pseudo-membranes of diphtheria and croup are, to all intents and purposes, identical; but it is held that the disease diphtheria is only one of several causes that may lead to croupous inflammation, be it in pharynx or larynx; in the mouth or rectum; in the pericardial cavity, or the cavity of the bladder; on the surfaces of the conjunctiva, the nasal mucous membranes,[1] the uterus, or the vagina; and this ground shall be defended, if need be, by authority, by *post mortem* reports, and by the history of bedside experiences.

Amongst the dangers of the parturient, that of septic infection has of late attracted perhaps the greatest attention.

The source of infection being doubtless in the genital canal, it is natural to suppose that the use of local antiseptic washes will do away with the cause of infection, and so irrigation of the vagina and uterus, either one or both, is practised, and, in a great majority of the cases, without an optical examination of the parts.

Now, it will occasionally happen that such irrigation is not followed by the prompt decline of symptoms expected. Then it may occur to the obstetrician that there exists a cause of trouble in some hidden corner of the uterus, and he irrigates the cavity of that organ vigorously and well. If, after this, there is no subsidence of fever and the other symptoms of infection, the obstetrician stands puzzled. Every member of this society, under similar circumstances, would avail himself of the benefit of a specular examination of the vagina, and, if no cause of sepsis was there discovered, would explore the uterus by the best instrumental means.

In a proportion of cases, the vagina, or the cervix uteri, would be found to be the seat of a croupous inflammation, the pseudo-membrane of which affording an excellent entrenchment for microörganisms, its fibrous structure opposing successfully the attack of the phenol or mercuric solution, becomes an undoubted source of septic infection, and the cause of all the trouble.

While believing that well-versed physicians would examine the vagina and discover such a croupous inflammation, it is not

1 Potter, F. H., Membranous Rhinitis. *Jour. Laryngology*, Mar., 1889, p. 89.

so certain that they would call it by its right name. Is it not true that most men would pronounce it a diphtheritic infection? Now, admittedly, this may be diphtheria, but, as a rule, it is a croupous inflammation of other origin. It is a focus of infection; it requires antiseptic and destructive treatment lest it lead to fatal septicemia; but in the progress of that septicemia there would not ordinarily be found the symptoms of diphtheria, nor would there be the danger of contagion which always belongs to that disease. The infant may be allowed to nurse the mother, in such cases, without fear of further disturbance than that which would naturally follow from nursing a woman suffering from general septicemia. The infant would not suffer from pseudo-membranous laryngitis, nor from diphtheria. And in the case of the affected mother, frequent viewing of the parts discloses that the changes occurring in the progress of the croupous inflammation, while showing a pseudo-membrane identical to that of diphtheria, are not so prone to extend to contiguous parts, not so intractible to local treatment, as the croupous inflammation of true diphtheria.

The object of this paper is to point out the not infrequent occurrence of such inflammations in the parturient genitalia; to state the true nature of this inflammation; to express a belief as to the dangers to the nursing child; and to offer an explanation why some cases of septic infection are not cured by the simple irrigation of the vagina and uterus with antiseptic washes.

278 Franklin Street.

---

The combination of medical journals has evidently been recognized as a wise movement. The *Medical Press of Western New York* and the Buffalo Medical and Surgical Journal have pooled their issues, and will hereafter appear under the latter name. Both have been publications of sterling merit, and it cannot be doubted that the result of the combination will be a better journal than either.—*Times and Register*.

